# Chromium deposition and poisoning of La_2_NiO_4_ cathode of solid oxide fuel cell

**DOI:** 10.1098/rsos.180634

**Published:** 2018-10-17

**Authors:** Bingxue Hou, Cheng Cheng Wang, Xumei Cui, Yungui Chen

**Affiliations:** 1 Department of Advanced Energy Materials, College of Materials Science and Engineering, Sichuan University, Chengdu 610065, People's Republic of China; 2 College of Vandium and Titanium, Panzhihua University, Panzhihua 617000, People's Republic of China; 3 The School of Entrepreneurship and Innovation, Shen Zhen Polytechnic, Shenzhen 518055, People's Republic of China

**Keywords:** chromium poisoning, La_2_NiO_4_, impedance spectroscopy, oxygen reduction, solid oxide fuel cell

## Abstract

Chromium deposition and poisoning of La_2_NiO_4_ cathode of solid oxide fuel cell were studied. La_2_NiO_4_ cathode showed stable performance in the presence of metallic interconnects. Comparing with the polarization resistance (*R*_p_) of La_2_NiO_4_ cathode in the absence of metallic interconnects, *R*_p_ did not change in the presence of metallic interconnect. After electrical conductivity relaxation method, La_2_NiO_4_ with high surface oxygen diffusion coefficients working under Cr atmosphere improved the oxygen reduction kinetics and increased cathode O_2_ reduction reaction rates. No chromium deposition was observed on the La_2_NiO_4_ cathode surface after polarization for 20 h at 800°C. The chemical compatibility of La_2_NiO_4_/Cr_2_O_3_ and La_2_NiO_4_/Gd_0.1_Ce_0.9_O_1.95_ (GDC) study indicates that La_2_NiO_4_ did not react with Cr_2_O_3_ and GDC under the operating temperature. The results indicate that La_2_NiO_4_ cathode is a potential chromium-tolerant material of solid oxide fuel cell.

## 1. Introduction

The intermediate-temperature (600–800°C) solid oxide fuel cells (SOFCs) is the high-efficiency and low-cost power generation system due to the feasibility of using less expensive metallic interconnect materials, such as chromia-forming alloys [1–3]. However, the deposition of Cr eventually leads to a serious polarization loss of the cathode and performance degradation of SOFCs [4–8]. The chromium deposition and poisoning on the cathode is becoming one of the most critical issues for the commercial viability and application of SOFC technologies [9,10]. Thus, development of cathodes with high resistance towards chromium poisoning becomes great challenge for the metallic interconnect based IT-SOFCs.

Until now, many researchers have already tried to develop several cathode materials to be tolerant towards chromium poisoning. Cheng *et al*. [11] studied the novel MIEC (La_0.24_Sr_0.16_Ba_0.6_) (Co_0.5_Fe_0.44_Nb_0.06_) O_3_ (LSBCFN) cathode by direct mixing synthesis of BCFN and LSCF powders. The results indicated that LSBCFN cathode showed an excellent stability and tolerance toward chromium deposition and poisoning under SOFC operation conditions. Zhen & Jiang [12] investigated that Sr-free (La,Ba)(Co,Fe)O_3_ had a potential to be a high performance cathode with high Cr-deposition resistance and Cr-tolerance for IT-SOFCs, in comparison with a (La,Sr)(Co,Fe)O_3_ (LSCF) electrode. Moreover, it had also been found that La(NiFe)O_3_-based cathodes had higher resistance to chromium poisoning [13,14]. Huang *et al.* [15] even reported that an LNF cathode impregnated with Gd_0.1_Ce_0.9_O_1.95_ (GDC; AGC Seimi Chemical Co. Ltd, Japan) made it possible to have good stability for long-term operation under Cr exposure due to very little Cr deposition.

Nowadays, of interest are Ni-based mixed conducting materials, e.g. the Ruddlesden–Popper phases with the K_2_NiF_4_-type structure. The mixed ionic–electronic conductor, La_2_NiO_4+_*_δ_* (LNO) is also being considered as a potential cathode material because of its high oxygen diffusion coefficients and good electronic conductivity [16–19]. Skinner & Kilner [17] showed that the oxygen tracer diffusion coefficient of LNO is higher than that of LSM and LSCF, which indicates also high ionic conductivity for LNO. Therefore, this Sr-free LNO cathode material will be paid much attention on the electrochemical performance, as well as stability in the future. In this paper, the fabrication and cathode behaviour of the LNO cathode have been investigated. The electrochemical performance of the LNO cathode for oxygen reduction is studied for the first time in the presence of chromium-forming alloy interconnects under the operating temperature.

## 2. Material and methods

Powders of La_2_NiO_4_ were prepared using a citrate–nitrate route described before [20]. After calcination at 950°C for 3 h, the as-prepared powders were confirmed by X-ray diffraction (XRD) to be single-phase, tetragonal La_2_NiO_4_. La_2_NiO_4_ powders were then pressed into a rectangular bar at 300 MPa and sintered at 1350°C for 4 h in air to form dense La_2_NiO_4_ bar with relative density higher than 96%, which was satisfied with the requirement of electrical conductivity relaxation (ECR) method [21]. The size of the sintered La_2_NiO_4_ bar samples had dimension of 25 mm × 6.6 mm × 0.62 mm. Measurement of oxygen surface exchange coefficient (*K*_chem_) was performed using ECR method. The effect of *in situ* chromium deposition and poisoning on the LNO bar samples were then studied at 800°C for a period of 24 h.

The polarization resistance (*R*_p_) of the La_2_NiO_4_ cathode was measured in the three-electrode cell configuration [22]. Electrolyte pellets were prepared by die pressing GDC powder, followed by sintering at 1500°C for 5 h. The pellets were 0.6–0.9 mm in thickness and 19–21 mm in diameter. Then La_2_NiO_4_ powders were dispersed in a solution of 20 wt% terpineol and 80 wt% cellulose to obtain the cathode ink, which was screen printed onto the electrolyte, followed by sintering in air at 1050°C for 3 h. The thickness of the La_2_NiO_4_ electrodes was 20–30 mm and the geometric area was 0.5 cm^2^.

A commercial Fe-Cr alloy RA446 (23–27% Cr, 1.5% Mn, 1% Si, 0.2% C, 0.12% N and the remaining Fe; Rolled Alloy Co., Ontario, Canada) was used as the interconnect materials. The alloys were machined into coupons (12 × 12 × 4 mm) with channels (1.2 × 1.2 mm) cut on one side of the coupons. Air was directed to the channels through an alumina tube. Two Pt wires were spot-welded to the coupon to serve as the voltage and current probes, respectively. The interconnect, which also acted as the current collector, was polished to expose the fresh surface and directly placed onto the surface of the La_2_NiO_4_ electrode. Detailed cell and interconnect configurations were reported elsewhere [23]. Electrochemical impedance responses were measured using a Gamry Reference 3000 Potentiostat. Impedance curves were recorded under open circuit with frequency range from 0.1 Hz to 100 kHz and the signal amplitude of 10 mV. The *R*_p_ was then measured by the differences between the high- and low-frequency intercepts. To determine the phase composition and chemical compatibility of La_2_NiO_4_ and GDC, Cr_2_O_3_, respectively, and pressed into pellets for XRD characterization at 1050°C, 800°C, respectively. The microstructure of the cells with and without chromium atmosphere was examined by scanning electron microscopy (SEM) and energy dispersive spectrometer using a Zesis EVO with 15 keV.

## 3. Results and discussion

### 3.1. XRD analysis

**Figure 1.**
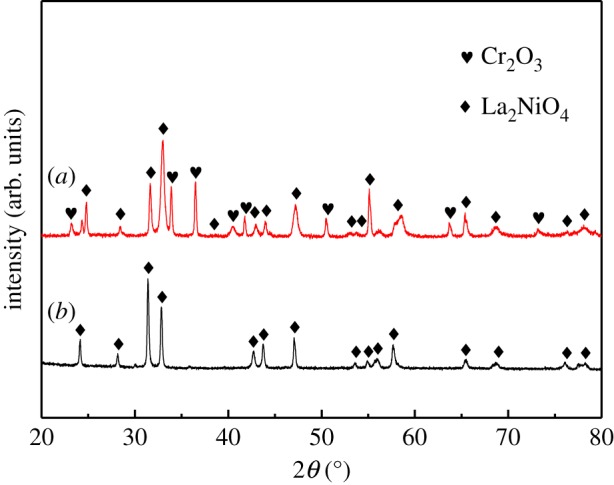
XRD patterns of La_2_NiO_4_ (*b*) and Cr_2_O_3_ powder mixtures (*a*) after heated at 800°C.

**Figure 2.**
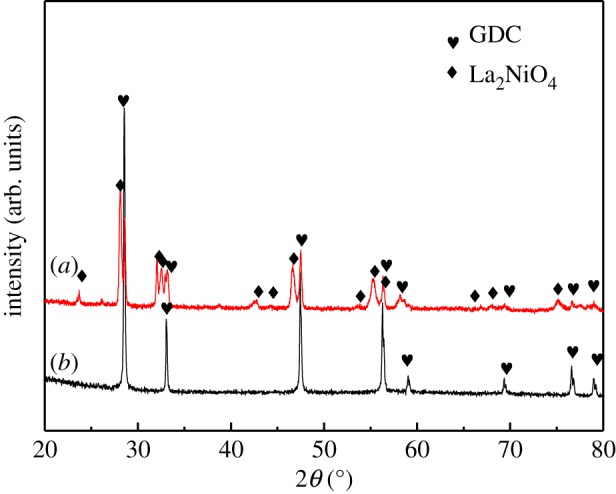
XRD patterns of GDC (*b*) and La_2_NiO_4_ powder mixtures (*a*) after heated at 1050°C.

Figure 1 shows XRD patterns of the La_2_NiO_4_ and La_2_NiO_4_/Cr_2_O_3_ mixture after heated at 800°C. It can be clearly seen from figure 1*b* that a single-phase, tetragonal La_2_NiO_4_(JCPDS PDF 00-034-0314) was formed and the result was in accordance with previous papers [24]. As shown in figure 1*a*, no new peaks were observed at 800°C. So it indicates that LNO and Cr_2_O_3_ did not react with each other at certain temperature. The results show that LNO could prevent from the deposition of chromium at 800°C. However, more works would still need to be done for the detailed understanding of the relationship between the LNO and chromium deposition in the future. No reaction or decomposition for LNO/CGO (JCPDS PDF 01-075-0162) was also observed by XRD for 24 h of treatment at 1050°C, as shown in figure 2. It also means that LNO prepared by citrate–nitrate method did not easily react with CGO at certain temperature, which was in accordance with the previous reported papers [25]. Other authors have also reported the compatibility of LNO with both YSZ and CGO at the mentioned temperatures for different periods of time [26,27].

### 3.2. Microstructure of LNO bar samples

**Figure 3.**
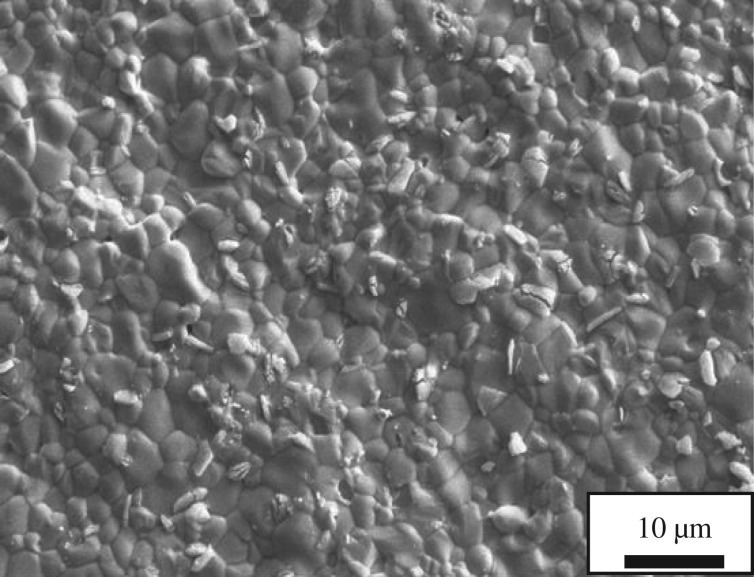
SEM micrographs of freshly prepared La_2_NiO_4_ surface.

**Figure 4.**
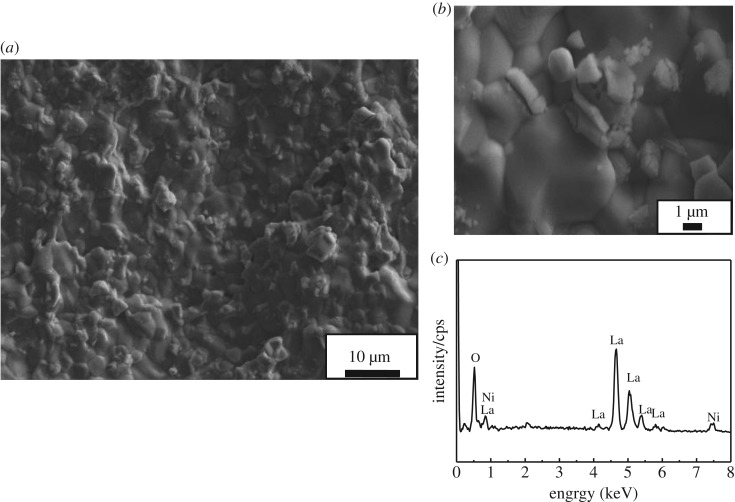
SEM and EDX of La_2_NiO_4_ surface heat-treated in the presence of Cr_2_O_3_ at 800°C for 24 h. (*a*) ×1000, (*b*) ×5000, (*c*) selected EDX points.

Figure 3 shows the SEM micrographs of LNO sintered at 1350°C. It can be clearly seen that the generally uniform and dense microstructure composed of well-grown grains around 1 µm was achieved at the sintering temperature of 1350°C. The specimen sintering at this temperature attains 96% of the theoretical density. Figure 4 shows SEM micrographs of the LNO surface heat treated in the presence of Cr_2_O_3_ for 24 h. It can be clearly seen that the size of the particles did not change quickly, comparing with the SEM micrograph without Cr_2_O_3_ of LNO fresh sample. Then the element distribution of selected point on the surface of LNO samples was examined by energy dispersion spectra (EDX) and all the results were given in figure 4*c*. It can be seen that for LNO bar samples treated at 800°C for 24 h in the presence of Cr, only La and Ni peaks were detected when they were measured on LNO grain surface. It indicates that these particles might be able to have a high tolerance towards chromium deposition; moreover, this result was also in accordance with the previous XRD result. Hildenbrand *et al.* [28] have reported that LNO was a potentially Cr-poisoning resistant cathode material.

### 3.3. ECR of LNO samples

**Figure 5.**
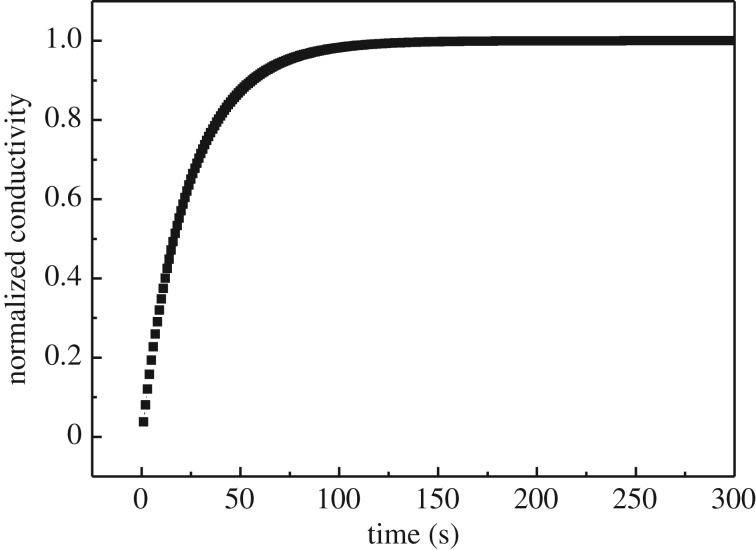
Normalized conductivity versus time at 800°C upon a step change of *P*_O2_ from 0.05 bar to 0.21 bar for the La_2_NiO_4_ cathode in the presence of Cr_2_O_3_.

Figure 5 shows typical normalized conductivity of LNO bar samples with Cr atmosphere at 800°C when the oxygen partial pressure changed from 0.05 bar to 0.21 bar. It can be clearly seen that the experimental data match well with the fitting curve. The relaxation time was about 100 s. After being exposed to the chromium for 24 h, the calculated *K*_chem_ and oxygen diffusion coefficient (*D*_chem_) of LNO at 800°C was 4 × 10^−3^ cm s^−1^ and 5 × 10^−4^ cm^2^ s^−1^, respectively. It shows that the surface exchange under such condition was fast and the overall relaxation process was already purely controlled by bulk diffusion of oxygen. There is still some literatures report about the fast surface exchange rate of LNO without Cr atmosphere [20]. It shows that the sample took 50 s and 150 s to reach equilibrium at 900°C and 750°C, respectively. *K*_chem_ and *D*_chem_ at 750°C were 3.9 × 10^−5^ cm^2^ s^−1^ and 1.6 × 10^−3^ cm s^−1^, respectively. It also points out that the fast surface exchange at high temperature was in good agreement with the literature on tracer and permeation measurements reporting higher activation energy for surface exchange than for diffusion in LNO [17,29]. It indicates that the ECR method used in this study was a reliable technique to study the exchange coefficient for LNO bar samples. Finally, it can also be concluded that there was no significant effect of the chromium atmosphere on the relaxation kinetics of LNO samples, which was in agreement with the previous EDX results.

**Figure 6.**
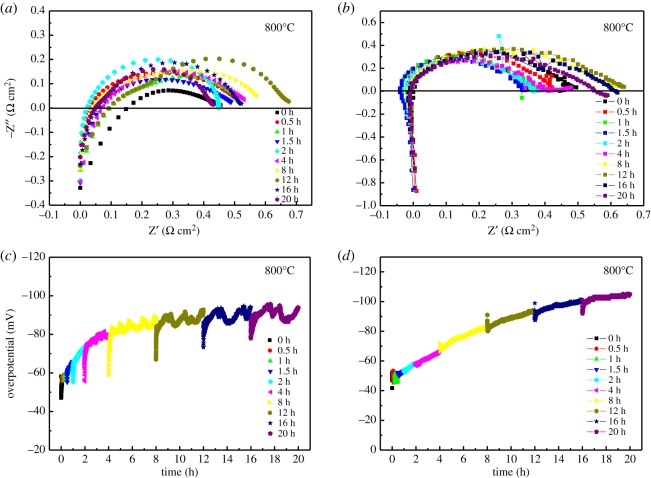
Polarization and impedance behaviour of La_2_NiO_4_ cathode in (*a* and *b*) the absence and (*c* and *d*) presence of metallic interconnects as a function of polarization time at 200 mA cm^−2^ for 20 h and 800°C. Impedance curves were measured at open circuit.

Figure 6 shows the electrochemical behaviour of LNO cathode under a current density of 200 mA cm^−2^ for 20 h at 800°C in the absence and presence of metallic interconnect. The electrolyte resistance had been subtracted from the cell impedance. It can be seen from figure 6*a,b*, the LNO electrode *R*_p_ increased slowly with the polarization time. The *R*_p_ of the LNO cathode under exposure of Cr for 20 h was nearly 0.6 Ω cm^2^, which was slightly higher than that of the same cathode after polarization for 0 h. Compared with the LNO cathode working in the absence of Fe–Cr alloy, the *R*_p_ did not change too much. Meanwhile, the varying trend of the overpotential (*η*) of LNO cathode working in the absence and presence of Fe–Cr alloy remained the same. *η* increased from 50 to 90 mV in the absence of Fe–Cr alloy, however, *η* increased from 50 to 100 mV in the presence of Fe–Cr alloy. So, this indicates that little effect on the chromium poisoning of the metallic interconnect on the activity and performance of LNO cathode. Sayers *et al.* [30] reported that the cathode area-specific resistance (ASR) of LNO working on CGO was 1.0 Ω cm^2^ at 700°C. What is more, Pérez-Coll *et al.* [31] studied that the cathode ASR of LNO working on Co doped, samarium doped ceria was 0.4 Ω cm^2^ at 800°C. It is commonly believed that the deposition process of (La,Sr)MnO_3_ and LSCF was related to the chemical dissociation of the gaseous Cr species, and is likely to be limited by the nucleation reaction between these Cr species and the nucleation agents (Mn^2+^) or Sr-segregation, and then it could lead to the increase in *R*_p_ and performance degradation [23]. Also, *R*_p_ increased significantly for BSCF cathode working in the same condition [32]. However, in this study the much lower increase in *R*_p_ for the O_2_ reduction reaction on the LNO cathode means that this Sr-free cathode material might not easily react with Cr species. It can be explained that for K_2_NiF_4_-type structure, LNO with high surface oxygen diffusion coefficients may improve the oxygen reduction kinetics and increase cathode O_2_ reduction reaction rates. Then LNO cathode might have a great potential in improving the ability of Cr-resistance in the future. More studies and explanations about SEM images of LNO cathode working in the presence of metallic interconnect under a current density of 200 mA cm^−2^ for 20 h will be discussed in detail.

**Figure 7.**
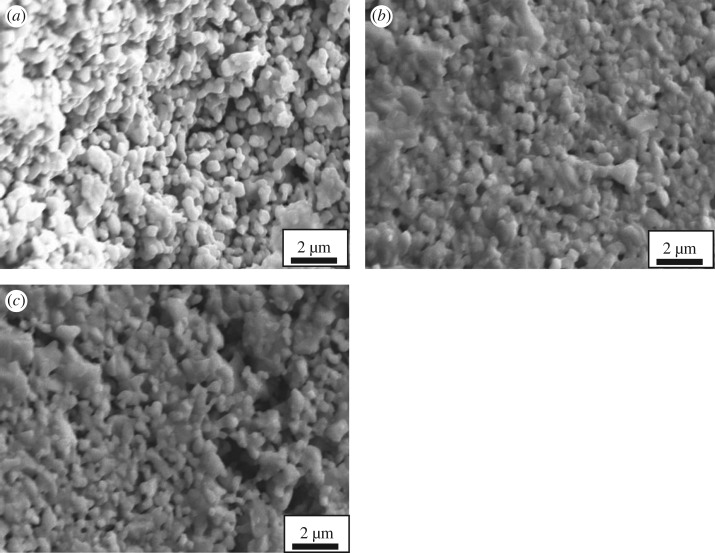
SEM micrographs of the surface of La_2_NiO_4_ cathode under rib and channels of the metallic interconnect after tested under a current density of 200 mA cm^−2^ for 20 h (*a* and *b*). The surface of a La_2_NiO_4_ cathode tested without interconnect is shown in (*c*).

**Figure 8.**
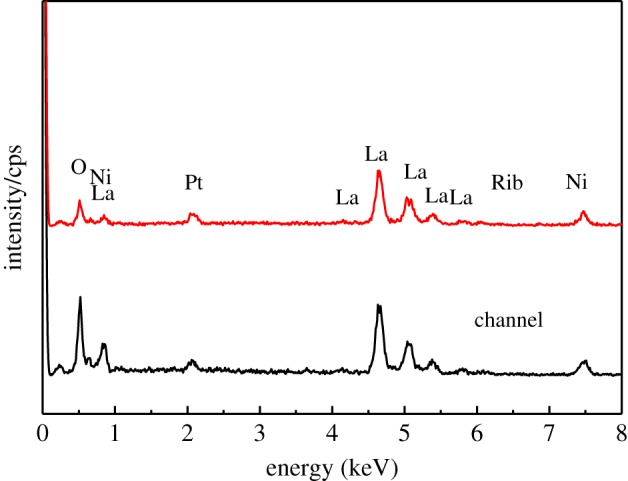
EDX spectra of the La_2_NiO_4_ cathode surface under rib and channel of the metallic interconnect tested after under a current density of 200 mA cm^−2^ for 20 h.

Figure 7 shows the SEM images of the cathode surface under the rib and channel of the metallic interconnect after 20 h current passage at 800°C. For the purpose of comparison, the SEM image of LNO cathode before Cr study is shown in figure 7*c*. The LNO cathode after current passage at 800°C both under the rib and channel of the interconnect was uniformly porous, and they were all similar to that observed on the surface of LNO cathode without Cr study. Figure 8 shows EDX spectra of the LNO cathode under the rib and channel of the metallic interconnect after 20 h current passage at 800°C. EDX analysis indicates that the absence of chromium deposits on the LNO electrode surface tested at 800°C. This is consistent with the SEM image result observed under the rib and channels of the metallic interconnect (figure 7). However, there were other reports about the chromium deposition on the LSCF cathode surface under the same conditions [33]. It clearly showed that the mechanism of the chromium deposition and poisoning at these cathodes was affected by the deposition process. LNO cathode material with Sr-free element could have a good tolerance towards chromium.

**Figure 9.**
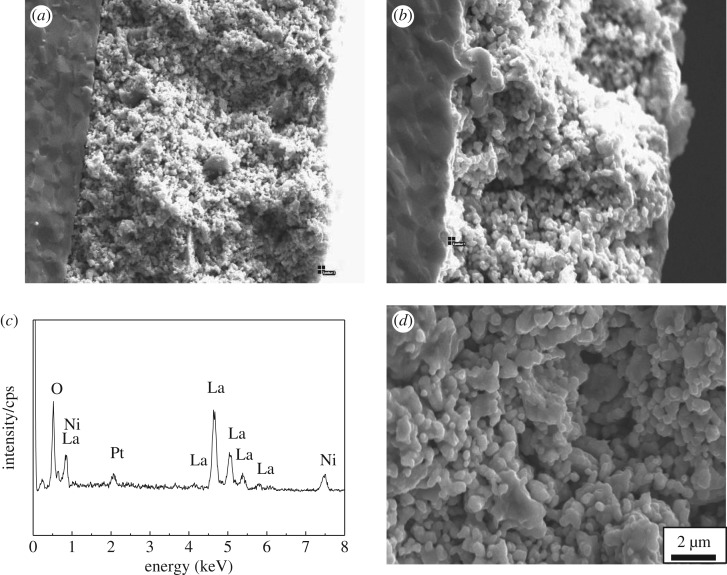
SEM micrographs of the fracture surfaces of La_2_NiO_4_ cathode in the presence (*a*) and absence (*b*) of metallic interconnect after tested under a current density of 200 mA cm^−2^ for 20 h, (*c*) selected points of EDX spectra of the La_2_NiO_4_ cathode in the presence of metallic interconnect, (*d*) porous La_2_NiO_4_ cathode.

Figure 9 shows SEM image of the LNO/GDC interface in the presence (*a*) and absence (*b*) of metallic interconnect after tested under a current density of 200 mA cm^−2^ for 20 h. It is obvious that GDC electrolyte thin film was essentially dense, with a continuous and crack free surface morphology and no pinholes. The porous structures were still kept in the presence of Cr atmosphere (figure 9*a*). Cr vapours in air were supplied inside the porous cathodes without significant microstructure change. The microstructures at the cathode/electrolyte interface show no voids and perfect adhesion of porous cathode structures on the GDC electrolyte. No Cr deposition was observed in the region of the LNO/GDC interface after operating under a current density of 200 mA cm^−2^ for 20 h and was confirmed by EDX (figure 9*c*). Evidently, the intensity of Cr at the LNO/GDC interface was low, which indicates that the deposition of Cr species at the interface was kinetically slow. From both the surface and interface images, we find that LNO cathode can reduce the Cr deposition. There are probably several reasons for this phenomenon. Firstly, this Sr-free LNO is K_2_NiF_4_-type structure, which consisted of perovskite layers of LaNiO_3_ separated by rock salt layers of LaO, with a network of unoccupied interstitial sites. So it also exhibits mixed electronic and ionic conductivity properties, which was highly desirable for expanding the reaction zone beyond three-phase boundaries [34]. Secondly, according to the results of LNO bar sample with Cr atmosphere tested at 800°C for 24 h, it clearly shows that LNO with high surface oxygen diffusion coefficients might improve the oxygen reduction kinetics and increase cathode O_2_ reduction reaction rates. Thirdly, some researchers have reported that LNO had higher ionic conductivity, comparing with LSCF and LSM [35–38]. So, it can improve the transport of oxygen ions from the cathode to the electrolyte. Therefore, LNO could probably become a Cr-resistance cathode material. It still needs further study about the stability of LNO cathodes operating in the presence of metal interconnect in the future.

## 4. Conclusion

Chromium deposition and poisoning of LNO cathode of SOFC are investigated. The *R*_p_ did not change too much with the polarization time at 800°C in the presence of metallic interconnect. After ECR method, LNO with high surface oxygen diffusion coefficients working under the Cr atmosphere may improve the oxygen reduction kinetics and increase cathode O_2_ reduction reaction rates. No Cr deposition is observed in the region of the LNO/GDC interface after operating under a current density of 200 mA cm^−2^ for 20 h. The chemical compatibility of LNO/Cr_2_O_3_ and LNO/GDC study indicates that LNO did not react with Cr_2_O_3_ and GDC under the operating temperature. The results demonstrate that La_2_NiO_4_ cathode is a potential chromium-tolerant material of SOFC.

## Data accessibility

This article does not have any additional data.

## References

[1] Inagaki T, Nishiwaki F, Yamasaki S, Akbay T, Hosoi K 2008 Intermediate temperature solid oxide fuel cell based on lanthanum gallate electrolyte. J. Power Sources 181, 274–280. (10.1016/j.jpowsour.2007.10.088)

[2] Jiang SP 2006 A review of wet impregnation—an alternative method for the fabrication of high performance and nano-structured electrodes of solid oxide fuel cells. Mater. Sci Eng. A 418, 199–210. (10.1016/j.msea.2005.11.052)

[3] Leng YJ, Chan SH, Jiang SP, Khor KA 2004 Low-temperature SOFC with thin film GDC electrolyte prepared in situ by solid-state reaction. Solid State Ionics 170, 9–15. (10.1016/j.ssi.2004.02.026)

[4] Badwal SPS, Deller R, Foger K, Ramprakash Y, Zhang JP 1997 Interaction between chromia forming alloy interconnects and air electrode of solid oxide fuel cells. Solid State Ionics 99, 297–310. (10.1016/S0167-2738(97)00247-6)

[5] Krumpelt M, Cruse TA, Ingram BJ, Roubort JL, Wang S, Salvador PA, Chen G 2010 The effect of chromium oxy-hydroxide on solid oxide fuel cells. J. Electrochem. Soc. 157, B228–B233. (10.1149/1.3266930)

[6] Taniguchi S, Kadowaki M, Kawamura H, Yasuo T, Akiyama Y, Miyake Y, Saitoh T 1995 Degradation phenomena in the cathode of a solid oxide fuel cell with an alloy separator. J. Power Sources 55, 73–79. (10.1016/0378-7753(94)02172-Y)

[7] Horita T, Xiong YP, Kishimoto H, Yamaji K, Brito ME, Yokokawa H 2010 Chromium poisoning and degradation at (La, Sr)MnO_3_ and (La, Sr)FeO_3_ cathodes for solid oxide fuel cells. J. Electrochem. Soc. 157, B614–B620. (10.1149/1.3322103)

[8] Chen X, Hua B, Pu J, Li J, Zhang L, Jiang SP 2009 Interaction between (La, Sr)MnO_3_ cathode and Ni-Mo-Cr metallic interconnect with suppressed chromium vaporization for solid oxide fuel cells. Int. J. Hydrogen Energ. 34, 5737–5748. (10.1016/j.ijhydene.2009.05.085)

[9] Jiang SP, Zhen Y 2008 Mechanism of Cr deposition and its application in the development of Cr-tolerant cathodes of solid oxide fuel cells. Solid State Ionics 179, 1459–1464. (10.1016/j.ssi.2008.01.006)

[10] Fergus JW 2009 Effect of cathode and electrolyte transport properties on chromium poisoning in solid oxide fuel cells. Cheminform 40, 3664–3671. (10.1016/j.ijhydene.2006.08.005)

[11] Chen XB, Jiang SP 2013 Highly active and stable (La_0.24_Sr_0.16_Ba_0.6_)(Co_0.5_Fe_0.44_Nb_0.06_)O_3−*δ*_ (LSBCFN) cathodes for solid oxide fuel cells prepared by a novel mixing synthesis method. J. Mater. Chem. A. 1, 4871–4878. (10.1039/c3ta10230k)

[12] Zhen Y, Jiang SP 2008 Characterization and performance of (La, Ba)(Co, Fe)O_3_ cathode for solid oxide fuel cells with iron-chromium metallic interconnect. J. Power Sources 180, 695–703. (10.1016/j.jpowsour.2008.02.093)

[13] Komatsu T, Arai H, Chiba R, Nozawa K, Arakawa M, Sato K 2006 Cr poisoning suppression in solid oxide fuel cells using LaNi(Fe)O_3_ electrodes. Electrochem. Solid-State Lett. 9, A9–A12. (10.1149/1.2130309)

[14] Zhen YD, Tok AIY, Jiang SP, Boey FYC 2007 La(Ni,Fe)O_3_ as a cathode material with high tolerance to chromium poisoning for solid oxide fuel cells. J. Power Sources 170, 61–66. (10.1016/j.jpowsour.2007.03.079)

[15] Huang B, Zhu XJ, Ren RX, Hu YX, Ding XY, Liu YB, Liu ZY 2012 Chromium poisoning and degradation at Gd_0.2_Ce_0.8_O_2_-impregnated LaNi_0.6_Fe_0.4_O_3−*δ*_ cathode for solid oxide fuel cell. J. Power Sources 216, 89–98. (10.1016/j.jpowsour.2012.05.058)

[16] Ganguly P, Rao CNR 1973 Electron transport properties of transition metal oxide systems with the KNiF structure. Mater. Res. Bull. 8, 405–412. (10.1016/0025-5408(73)90044-5)

[17] Skinner SJ, Kilner JA 2000 Oxygen diffusion and surface exchange in La_2−*x*_Sr_*x*_NiO_4+*δ*_. Solid State Ionics 135, 709–712. (10.1016/S0167-2738(00)00388-X)

[18] Emmanuelle B, Bassat J-M, Dordor P, Mauvy F, Grenier JC, Stevens P 2005 Oxygen diffusion and transport properties in non-stoichiometric Ln_2−*x*_NiO_4+*δ*_ oxides. Solid State Ionics 176, 2717–2725. (10.1016/j.ssi.2005.06.033)

[19] Bassat JM, Odier P, Loup JP 1994 The semiconductor-to-metal transition in question in La_2−*x*_NiO_4+*δ*_(*δ* > 0 or *δ* < 0). J. Solid State Chem. 110, 124–135. (10.1006/jssc.1994.1146)

[20] Li Z, Haugsrud R 2012 Effects of surface coatings on the determination of *D*_chem_ and *k*_chem_ in La_2_NiO_4+*δ*_ by conductivity relaxation. Solid State Ionics 206, 67–71. (10.1016/j.ssi.2011.11.011)

[21] Zhao L, Hyodo J, Chen K, Ai N, Amarasinghe S, Ishihara T, Jiang SP 2013 Effect of boron deposition and poisoning on the surface exchange properties of LSCF electrode materials of solid oxide fuel cells. J. Electrochem. Soc. 160, F682–F686. (10.1149/2.131306jes)

[22] Jiang SP, Zhang JP, Apateanu L, Foger K 1999 Deposition of chromium species on Sr-doped LaMnO_3_ cathodes in solid oxide fuel cells. Electrochem. Commun. 1, 394–397. (10.1016/S1388-2481(99)00080-6)

[23] Jiang SP, Zhang JP, Apateanu L, Foger K 2000 Deposition of chromium species at Sr-doped LaMnO_3_ electrodes in solid oxide fuel cells. I. Mechanism and kinetics. J. Electrochem. Soc. 147, 4013–4022. (10.1149/1.1394012)

[24] Efimov K, Arnold M, Martynczuk J, Feldhoff A 2009 Crystalline intermediate phases in the sol-gel-based synthesis of La_2_NiO_4+*δ*_. J. Am. Ceram. Soc. 92, 876–880. (10.1111/j.1551-2916.2009.02943.x)

[25] Montenegro-Hernández A, Vega-Castillo J, Mogni L, Caneiro A 2011 Thermal stability of Ln_2_NiO_4+*δ*_(Ln: La, Pr, Nd) and their chemical compatibility with YSZ and CGO solid electrolytes. Int. J. Hydrogen Energ. 36, 15 704–15 714. (10.1016/j.ijhydene.2011.08.105)

[26] Sayers R, Liu J, Rustumji B, Skinner SJ 2008 Novel K_2_NiF_4_-type materials for solid oxide fuel cells: compatibility with electrolytes in the intermediate temperature range. Fuel Cells 8, 338–343. (10.1002/fuce.200800023)

[27] Zhao H, Mauvy F, Lalanne C, Bassat JM, Fourcade S, Grenier JC 2008 New cathode materials for ITSOFC: phase stability, oxygen exchange and cathode properties of La_2−*x*_NiO_4+*δ*_. Solid State Ionics 179, 2000–2005. (10.1016/j.ssi.2008.06.019)

[28] Hildenbrand N, Nammensma P, Blank DHA, Bouwmeester HJM, Boukamp BA 2013 Influence of configuration and microstructure on performance of La_2_NiO_4+*δ*_ intermediate-temperature solid oxide fuel cells cathodes. J. Power Sources 238, 442–453. (10.1016/j.jpowsour.2013.03.192)

[29] Smith JB, Norby T 2006 On the steady-state oxygen permeation through La_2_NiO_4+*δ*_ membranes. J. Electrochem. Soc. 153, A233–A238. (10.1149/1.2138679)

[30] Sayers R, Rieu M, Lenormand P, Ansart F, Kilner JA, Skinner SJ 2011 Development of lanthanum nickelate as a cathode for use in intermediate temperature solid oxide fuel cells. Solid State Ionics 192, 531–534. (10.1016/j.ssi.2010.02.014)

[31] Pérez-Coll D, Aguadero A, Escudero MJ, Núñez P, Daza L 2008 Optimization of the interface polarization of the La_2_NiO_4_-based cathode working with the Ce_1–*x*_Sm_*x*_O_2–*δ*_ electrolyte system. J. Power Sources 178, 151–162. (10.1016/j.jpowsour.2007.12.030)

[32] Kim YM, Chen XB, Jiang SP, Bae J 2011 Chromium deposition and poisoning at Ba_0.5_Sr_0.5_Co_0.8_Fe_0.2_O_3−*δ*_ cathode of solid oxide fuel cells. Electrochem. Solid-State Lett. 14, B41–B45. (10.1149/1.3549169)

[33] Chen XB, Zhang L, Jiang SP 2008 Chromium deposition and poisoning on (La_0.6_Sr_0.4−*x*_Ba_*x*_)(Co_0.2_Fe_0.8_)O_3_(0≤*x*≤0.4) cathodes of solid oxide fuel cells. J. Electrochem. Soc. 155, B1093–B1101. (10.1149/1.2969914)

[34] Kleitz M, Petitbon F 1996 Optimized SOFC electrode microstructure. Solid State Ionics 92, 65–74. (10.1016/S0167-2738(96)00464-X)

[35] Kim G, Wang S, Jacobson AJ, Chen CL 2006 Measurement of oxygen transport kinetics in epitaxial La_2_NiO_4+*δ*_ thin films by electrical conductivity relaxation. Solid State Ionics 177, 1461–1467. (10.1016/j.ssi.2006.07.013)

[36] Shaula AL, Naumovich EN, Viskup AP, Pankov VV, Kovalevsky AV, Kharton VV 2009 Oxygen transport in La_2_NiO_4+*δ*_: assessment of surface limitations and multilayer membrane architectures. Solid State Ionics 180, 812–816. (10.1016/j.ssi.2009.01.005)

[37] Adler SB 1996 Electrode kinetics of porous mixed-conducting oxygen electrodes. J. Electrochem. Soc. 143, 1881–1884. (10.1149/1.1837252)

[38] Xu Q, Huang DP, Zhang F, Chen W, Chen M, Liu HX 2008 Structure, electrical conducting and thermal expansion properties of La_0.6_Sr_0.4_Co_0.8_Fe_0.2_O_3−δ_-Ce_0.8_Sm_0.2_O_2−*δ*_ composite cathodes. J. Alloys Compounds 454, 460–465. (10.1016/j.jallcom.2006.12.132)

